# Wild *Leporinus friderici* induced spawning with different dose of mGnRHa and metoclopramide or carp pituitary extract

**DOI:** 10.21451/1984-3143-AR2019-0078

**Published:** 2020-02-20

**Authors:** Thiago Gonçalves de Souza, Rafael Yutaka Kuradomi, Suely Marlene Rodrigues, Sergio Ricardo Batlouni

**Affiliations:** 1 Centro de Aquicultura, Universidade Estadual Paulista, Jaboticabal, SP, Brasil; 2 Instituto de Ciências Exatas e Tecnologia, Universidade Federal do Amazonas, Itacoatiara, AM, Brasil; 3 Piscicultura Projeto Peixes, Sales de Oliveira, SP, Brasil

**Keywords:** Linpe method, ovulation, reproduction

## Abstract

Breeding technology is of utmost importance for reproduction of wild fish in captivity for the reintroduction and selective breeding programs purposes. The main challenge is that when applied to wild undomesticated specimens, conventional protocols often cause breeders and/or embryo mortality and spawning failure. In this study, we evaluated the reproductive performance of wild *Leporinus friderici*, a great importance fish for subsistence fishing in South American rivers, applying conventional and lower-dose hormonal therapies by means of two consecutive experiments. In the first, a conventional (0.5 and 5.5 mg/kg) and a lower carp pituitary extract (CPE) dose (0.5 and 1.0 mg/kg) were applied. In the second, a conventional mammalian GnRH analogue associated with metoclopramide (mGnRHa + MET) (40 µg mGnRHa + 20 mg MET/kg) and a lower dose (4 μg mGnRHa + 2 mg MET/kg and 8 µg + 4 mg of mGnRHa + MET/kg) were applied. Ovulation was observed in all treatments, however, only lower CPE protocol provided viable embryos. High levels of 17α,20β-dihydroxy-4-pregnen-3-one (DHP) and 17β estradiol **(**E_2_) detected in conventional, but not in lower CPE dose, at ovulation, might be associated to the mortality of the embryos. The use of lower CPE dose applied here was the best way to obtain *L. friderici* viable embryos. These results directly contribute to the knowledge about poorly explored effects of reproductive management and hormonal therapies in wild-type breeders, showing that the use of reduced doses may be an alternative to reproductive success.

## Introduction

*Leporinus friderici* is a medium size reophilic characiform fish native from South American rivers (Lopes and Leal, 2010). The species is one of great importance for subsistence fishing (Brasil, 2014), as it is widely used in repopulation programs caused by impacts where there are hydroelectric dams. Besides that, the species, together with other congeners, appears in official data production statistics, among the most produced fish in Brazil in 2018 (IBGE, 2018), mainly by presenting high quality meat and acceptance for commercial and sport fishing (Vaz et al., 2000) purposes. According to the same report (IBGE, 2018), the group of fish known as piaus or piavas, which includes *L. friderici*, maintained a stable production between 2017 (3,801T) and 2018 (3,080 T). Although it is relatively recurrent in South American rivers, there are reports of reduction of natural stocks due to environmental degradation and predatory fishing (Andrade et al., 2005).

In this context, one of the bottlenecks for the consolidation of its production in captivity, for aquaculture and preservation purposes, is the attainment of viable embryos through hormonal induction. When kept in captivity, ovarian and testicular maturation takes place, reaching more advanced stages of maturation between November and February (rainy season in Brazil) and, as well as the majority of tropical reophilic fish, hormonal stimulation is required for spawning (Borella et al., 2019; Hainfellner et al., 2019).

Still in this context, the most commonly protocol used for obtaining viable embryos in South American reophilic species is still the use of carp pituitary extract (CPE) in conventional dose (0.5 and 5.0 mg/kg), which is applied in a generalized way in different species (Carneiro and Mikos, 2008; Caneppele et al., 2015; Souza et al., 2015; Ittzés et al., 2015; Viveiros et al., 2015; Schorer et al., 2016; Pereira et al., 2017). However, the main problem related to the use of CPE is a constant uncertainty and unpredictability of a successful ovulation (Criscuolo-Urbinati et al., 2012; Hainfellner et al., 2012a, b; Pereira et al., 2017). In the specific case of *L. friderici*, the lower potential to induce ovulation, the heterogenous results concerning fertility rates and the number of oocytes that are retained in the post-spawning ovaries using CPE is highlighted by Sato et al. (2000). A high proportion of oocytes retained in the ovaries after stripping (week ovulation) seems also to be a constant in treatments using CPE in diverse South American reophilic species (Hainfellner et al., 2012a, b; Sato et al., 2000; Criscuolo-Urbinati et al., 2012; Pereira et al., 2017; Kuradomi and Batlouni, 2018).

Parallelly, the use of Gonadotrophin-releasing hormones analogs (GnRHa) has increased rapidly because of many advantages, especially because they are not species-specific molecules, but having high structural similarities among fish. Moreover, due to their synthetic nature, they have no risk of transmitting diseases such as CPE and, since they act at higher levels of the hypothalamic-pituitary gonad axis, they stimulate the release of endogenous LH and FSH, as well as other pituitary hormones that may have important reproductive functions (review in Mylonas et al., 2010). However, although it has already been shown to be efficient for provoking ovulation and viable embryos for few South American reophilic species (Ittzés et al., 2015; Viveiros et al., 2015; Souza et al., 2018), the use of GnRH (with or without dopamine inhibitors) is frequently associated with ovulation failures (Ramos et al., 1997; Carneiro and Mikos, 2008) and/or death of embryos (Acuña and Rangel, 2009; Paulino et al., 2011; Pereira et al., 2017) in this species, even when conventional worldwide used dose is applied. We also emphasize the importance of knowing and considering the largely unexplored impacts of management on the reproductive performance of wild specimens, which certainly interfere directly with reproductive performance (Bobe and Labbé, 2010). Thus, in this study we aimed to obtain an efficient and safety protocol for *L. friderici* induced breeding using different CPE or mGnRHa + MET protocols. The definition of protocols applied were based on data available in literature, enabling a comparison of reproductive performance and evolution of the meiotic evolution among different applied protocols.

## Methods

### Wild breeders capture and maintenance

Wild *L. friderici* broodstock was collected on fish passage ladders, of Small Hydropowers, located in the Sapucai Mirim River, São Joaquim da Barra, São Paulo, Brazil (-20.494067, -47.859124). Captured fish were transferred to Aquaculture Center of UNESP - CAUNESP (Jaboticabal, SP) and for the “Projeto Peixes” fish farming (Sales de Oliveira, SP). Breeders were acclimated (for three months), domesticated for 2 years and marked with microchips AnimallTAG^®^ (Korth RFID Ltda, São Carlos, SP). After that, fish were kept in earthen ponds of 300 m^3^ (20 m long × 10 m wide × 1.5 m deep) at a density of ~ 0.2 fish/m^3^, fed to satiety six days a week, in two, at 8:00 and 17:00, with a commercial extruded diet for omnivores (composition: 12.0% moisture content; 32.0% crude protein, 4.5% ether extract, 9.0% fiber, 3.5% calcium, 6.0% phosphorus). These breeders are part of an ongoing river fish repopulation project, used for the accomplishment of genetically directed crosses that aim to produce fingerlings of some species, including *L. friderici*, preserving the genetic variability of these species existing in the Sapucaí Mirim River (São Paulo, Brazil).

During domestication water parameters were measured weekly using oximeter HI 9146-10 (Hanna instruments) to determine dissolved oxygen, a pHmeter HI 98172 (Hanna Instruments) to pH and HI 98311 (Hanna Instruments) apparatus to electrical conductivity of the water and temperature.

### Induced reproduction

During *L. friderici* breeding season, at the time of spawning, broodstock fish were transported to the laboratory for acclimatization and maintained at the laboratory to conduct two experiments. The experiments were conducted in a semi-natural system. To that, five water tanks with a total volume of 750 L (filled with approximately 400 L of water) were used for each treatment containing two males and two females randomly distributed and each fish was considered as an experimental unit (Table 1).

**Table 1:** Experimental design used in this study for *Leporinus friderici* induced spawning.

**Experiment**	**Treatment**	**Water boxes (nº)**	**Male: Females**	**Total (nº)**	
			**Ratio/Box**	**Female**	**Male**
Experiment 1	Conventional dose	5	2: 2	10	10
CPE	Low dose	5	2: 2	10	10
Experiment 2 mGnRHa + MET	Conventional dose	5	2: 2	10	10
	Low dose	3	2: 2	6	6

CPE: carp pituitary extract. Conventional doses: 0.5 and 5.5 mg/kg (12 hour interval) and low doses: 0.5 and 1.0 mg/kg (6 hour interval); mGnRHa + MET: mammalian analogue gonadotrophin-releasing hormone associated with metoclopramide. Conventional dose: 40 μg mGnRHa + 20 mg MET/kg (single dose) and low dose: 4 μg mGnRHa + 2 mg MET/kg and 8 μg mGnRHa and 4 mg MET/kg (interval between doses of 6 hours).

The study was conducted by means of two simultaneous experiments. In the first experiment, we compared both the reproductive performance using the conventional (0.5 mg and 5.5 mg/kg) and a lower (0.5 mg and 1.0 mg/kg) dose (Table 2). The conventional CPE dose is used with some variations in intervals and concentrations among several studies, and we opted for 0.5 mg and 5.5 mg/kg, with 12h interval. The reduced dose of CPE applied was based on a study published with another tropical species *Schizodon fasciatus* (0.5 mg and 1.0 mg/kg, with a six-hour interval) which provided ovulation and obtaining viable embryos (Lopes and Leal, 2010).

**Table 2:** Experimental design used for *Leporinus friderici* females induced spawning in this study.

**Experiment**	**Treatment**	**Applied doses/kg**		
		**1^st^ dose**	**Interval (h)**	**2^nd^ dose**
Experiment 1	Conventional dose	0.5 mg	12	5.5 mg
CPE	Low dose	0.5 mg	6	1.0 mg
Experiment 2	Conventional dose	40 µg + 20 mg	-	-
mGnRHa + MET	Low dose	4 µg + 2 mg	6	8 µg + 4 mg

CPE: carp pituitary extract; mGnRHa + MET: mammalian analogue gonadotrophin-releasing hormone associated with metoclopramide.

In the second experiment, a single dose of 40 μg mammalian gonadotrophin-releasing hormone analogue (mGnRHa) + 20 mg metoclopramide (MET)/kg (determined as “conventional dose” for its wide use (Mylonas et al., 2010) and a lower fractioned dose (4 μg mGnRHa + 2 mg MET/kg and 8 µg mGnRHa + 4 mg MET/kg) (Table 2). The reduced dose of mGnRHa was based on a recent study with a congener species, *Leporinus macrocephalus* (Pereira et al., 2017) and on another study published with *Tinca tinca* (Podhorec et al., 2011) in which lower dose (between 1-20 µg mGnRH) provided successful ovulation. For all treatments, males were injected with a single dose of CPE (at the concentration of 1.0 mg/kg) at the time of female’s single or second dose.

We emphasize that the number of wild breeders authorized to be collected in this project by environmental agencies in São Paulo State, Brazil, did not include fish enough to perform control groups treated only with saline solution. Furthermore, because of the risk of loss of the scarce wild breeders during the hormonal induction procedure and because it is widely and for decades known that breeders of this and most rheophilic fish do not reproduce without hormonal induction (for review see Von Ihering and Azevedo, 1936; Nagahama and Yamashita, 2008; Bobe and Labbé, 2010; Mylonas et al., 2010; Borella et al., 2014, 2019), we opted to not use saline controls.

The CPE used in this study was the Stoller Fisheries brand (Spirit Lake, Iowa, USA). The mGnRHa + MET used was of the Ovopel^®^ brand (Interfish Ltd, Budapest, Hungary), whose GnRHa molecule has the D-Ala6, Pro9-Net modifications in the amino acid sequence. Each Ovopel^®^ pellet contained 18-20 μg mGnRHa and 8-10 mg metoclopramide (Cejko et al., 2012). The hormones used were diluted in saline solution (0.9%) and applied to the ventral muscles. The volume injected, regardless of the concentration of each dose, was 0.5 mL/kg.

### Reproductive performance evaluation

The latency period was defined as the time between the second or single injection and fish ovulation. To that, we determined the accumulated thermal units (ATU) interval between the second or single hormonal dose and spawning. ATU was calculated as the sum of the water temperature (°C) over time (hours) after the second or single hormonal dose.

For evaluating reproductive performance in each experiment, we compared the spawning rate (SR) (number of spawning females/total number of injected females × 100). The relative fecundity (RF) (number of eggs released per gram of fish) was also determined. To that, the total number of oocytes obtained in each experimental unit (absolute fecundity) was first estimated. To analyse this data, the total mass of eggs in each experimental unit was recorded. Sub-samples (~1 g) of the egg mass were used to extrapolate the total egg numbers. To determine relative fecundity, the sum of the body mass of the two females of each tank was used in the denominator.

Soon after spawning, 20 mL of hydrated eggs of each spawned female were carefully transferred to a 7 L funnel type conical incubator with a constant water flow. To determine the fertilization success (FS) of the eggs, 8-12 h post-fertilization (hpf) (after the blastopore closure stage), 100 eggs from each female were randomly sampled and counted, and those which were normally dividing were scored. After 17 hpf, overall hatching success (HS) was determined by counting the number of hatched eggs/number of fertilized eggs ×100. Values were determined for each female and after an average was calculated.

### Stereological evaluation of ovaries collected immediately after spawning

For histologic evaluation (volume density), five randomly collected spawned females for each treatment were euthanized with a lethal dose of benzocaine (2 g/L) at the time of ovulation. The cranial, medial and tail regions of the ovary tissues were fixed in 2.5% Glutaraldehyde solution for 24 hours and processed following routine histologic procedures, embedded in glycol methacrylate resin (Leica historesin embedding kit, Leica Microsystems, Nussloch, DE) for histologic preparation and stained with hematoxylin-floxin.

Different stages of development were classified by histological characteristics. We used a similar methodology applied by Criscuolo-Urbinati et al. (2012) and Pereira et al. (2017), since the morphology of the cells were similar and comparable among these migratory species.

### Determination of oocyte cell diameter

For the description of oocyte diameter, 20 oocytes (by female) from each phase [previtellogenic oocyte (PV); cortical alveoli oocyte (CA); complete vitellogenic (CV); mature vitellogenic oocytes with cytoplasm filled entirely by yolk and showing germinal vesicle break down (GVBD oocytes) and atretic oocyte (AT)] were considered. For each oocyte, two measurements of diameter were recorded, using ovaries collected for stereological evaluation. Finally, the mean of all oocytes recorded in each phase was determined. Measurements were performed with the aid of a microscopic Leica DM4000 binocular (Leica Microsystems, Wetzlar, Germany), equipped with Leica LAS v4.3.0 software (Leica Microsystems).

### Stereological evaluation

For this analysis only the females that ovulated in all treatments were used. All the ovulated females were analysed. Volume density occupied by different ovarian structures was determined using light microscopy and a 352 - intersection grid. To that, 4 microscopic fields from medial region of the ovary were randomly obtained, with a total of 1408 points scored for each animal with a 5x objective. The points on the classified structures were computed and their frequencies calculated (n° points * 100/total points), similar to the method used by Criscuolo-Urbinati et al. (2012) and Pereira et al. (2017). Points were classified as one of the following: previtellogenic oocyte (PV), cortical alveoli oocyte (CA), complete vitellogenic (CV), mature vitellogenic oocytes with cytoplasm filled entirely by yolk and showing germinal vesicle break down (GVBD oocytes) and atretic oocyte (AT). The appearance of post-ovulatory follicles (POF) and interstitial tissue (IT) were also characterized. Artefacts were rarely observed and were not considered in the total number of points used to obtain the percentages.

### Blood sampling and steroids assays

Blood was collected at the time of first (or single) hormonal dose and at the time of ovulation (all females were sampled). Animals were anesthetized with benzocaine (9 mg/L) for blood sampling. Blood was collected by puncturing the caudal vein with heparinized syringes (Liquemine, Roche, Rio de Janeiro, RJ, Brazil) and needles. Blood was centrifuged at 1300 g for 10 min. The plasma was separated into aliquots and frozen at -80 °C for the subsequent 17β estradiol (E_2_) and 17α,20β-dihydroxy-4-pregnen-3-one (DHP) assay. The plasma steroid level was measured by enzyme-linked immunosorbent assay (ELISA) E_2_ (DRG Instruments GmbH, Marburg, HE, DE) and DHP (Cayman Chemical Company, Ann Arbor, MI, EUA). Plasma samples were run in duplicate with an acceptable limit of ≤ 20.0 for the intra-assay coefficients of variation (Brown et al., 2004). The absorbance measurements were performed in a microplate reader (Epoch 2, Biotek Instruments Inc., Winooski, VT, USA).

### Certification

This work was certified, and documented with protocol number 019375/13, in accordance with ethical principles in animal experimentation. The certification was adopted by the National Council for Control of Animal Experimentation (CONCEA) and approved by the Committee on Ethics in Animal Use (CEUA), of the Paulista State University “Júlio de Mesquita Filho” of Jaboticabal, SP.

### Analysis of results

Statistical analysis was performed using the STATISTICA 7.0 computer program (StatSoft, Inc., Tulsa, OK, USA). Assumptions such as normality and homoscedasticity were tested. Variables such as body weight, standard length and relative fecundity rate were analysed using Student's t-test. Variables such as fertilization rate and volume density were analysed using the Mann-Whitney U test. The tests were performed with significance level of α = 0.05, with data expressed as mean followed by standard error (mean ± SEM).

## Results

### Sampling and water parameters

For both experiments, the mean body weight (g) and standard length (cm) of fish were similar between treatments (p > 0.05) (Table 3). Water parameters were: dissolved oxygen (6.2 ± 0.3 mg/L), pH (7.0 ± 1.0), temperature (23.0 ± 1.2 °C) and electrical conductivity of the water (31.7 ± 2.3 µS/cm).

**Table 3:** Weight (g) and standard length of *Leporinus friderici* breeders used in this study.

**Experiment**	**Treatment**	**Females**			**Males**	
		**Weight (g)**		**Standard lenght (cm)**	**Weight (g)**	**Standard lenght (cm)**
Experiment 1	Conventional dose	803.0 ± 66.1 A	30.9 ± 0.6 A		293.5 ± 43.1 A	23.4 ± 0.9 A
CPE	Low dose	656.7 ± 37.1 A	31.4 ± 0.4 A		291.9 ± 18.3 A	23.6 ± 0.6 A
Experiment 2	Conventional dose	625.5 ± 86.1 A	29.3 ± 1.1 A		270.5 ± 27.0 A	23.0 ± 0.7 A
mGnRHa + MET	Low dose	458.2 ± 34.4 A	28.0 ± 0.9 A		263.0 ± 18.2 A	23.8 ± 0.4 A

CPE: carp pituitary extract; mGnRHa + MET: mammalian analogue gonadotrophin releasing hormone associated with metoclopramide. Values are presented with mean followed by standard error (mean ± SEM). Different capital letters indicate statistical difference (p < 0.05) between different treatments of the same experiment.

### Induced breeding and reproductive performance

#### Experiment 1

The latency period was higher for conventional CPE dose than for lower dose. It was not possible to statically compare the SR between the treatments, but the values were relatively high for both conventional (80%) and lower CPE dose (100%). Relative fecundity was similar between treatments and the main differences between conventional and lower dose were detected with respect to the FS and HS (Table 4). The treatment with conventional CPE dose did not provide viable embryos (Table 4).

**Table 4:** *Leporinus friderici* reproductive performance of females submitted to two experiments for induced spawning using CPE, experiment “1”, and mGnRHa + MET, experiment “2”.

**Experiment**	**Treatment**	**ATU**	**SR (%)**	**RF**	**FS (%)**	**HS (%)**
Experiment 1	Conventional dose	254.0 ± 6.9 A	80	110.4 ± 21.2 A	01.2 ± 0.9 A	0
CPE	Low dose	153.5 ± 6.6 B	100	146.8 ± 26.1 A	74.9 ± 8.3 B	85.0 ± 5.8
Experiment 2	Conventional dose	258.1 ± 4.0 A	70	059.4 ± 21.5 A	00.8 ± 0.5 A	0
mGnRHa + MET	Low dose	158.6 ± 0.0 B	50	052.9 ± 23.9 A	00.9 ± 0.0 A	0

CPE: carp pituitary extract. mGnRHa + MET: mammalian analogue gonadotrophin-releasing hormone associated with metoclopramide. Values are presented with mean followed by standard error (mean ± SEM). Different capital letters indicate statistical difference (p < 0.05) between different treatments of the same experiment. ATU: “accumulated thermal units” between the first or single injection and fish ovulation. Calculated as the sum of the water temperature (°C) over time (hours) after the second dose; SR: spawning rates; RF: relative fertility rate (number of eggs/gram of fish); FS: fertilization success; HS: hatching success.

#### Experiment 2

The latency period was higher for mGnRHa + MET conventional dose than for lower dose. The SR were 70% and 50% respectively for mGnRHa + MET conventional and lower dose. Relative fecundity, FS and HS was similar between mGnRHa + MET treatments. For both mGnRHa + MET treatments, FS and HS were zero or very close to zero (Table 4).

### Stereological evaluation

**Figure 1:**
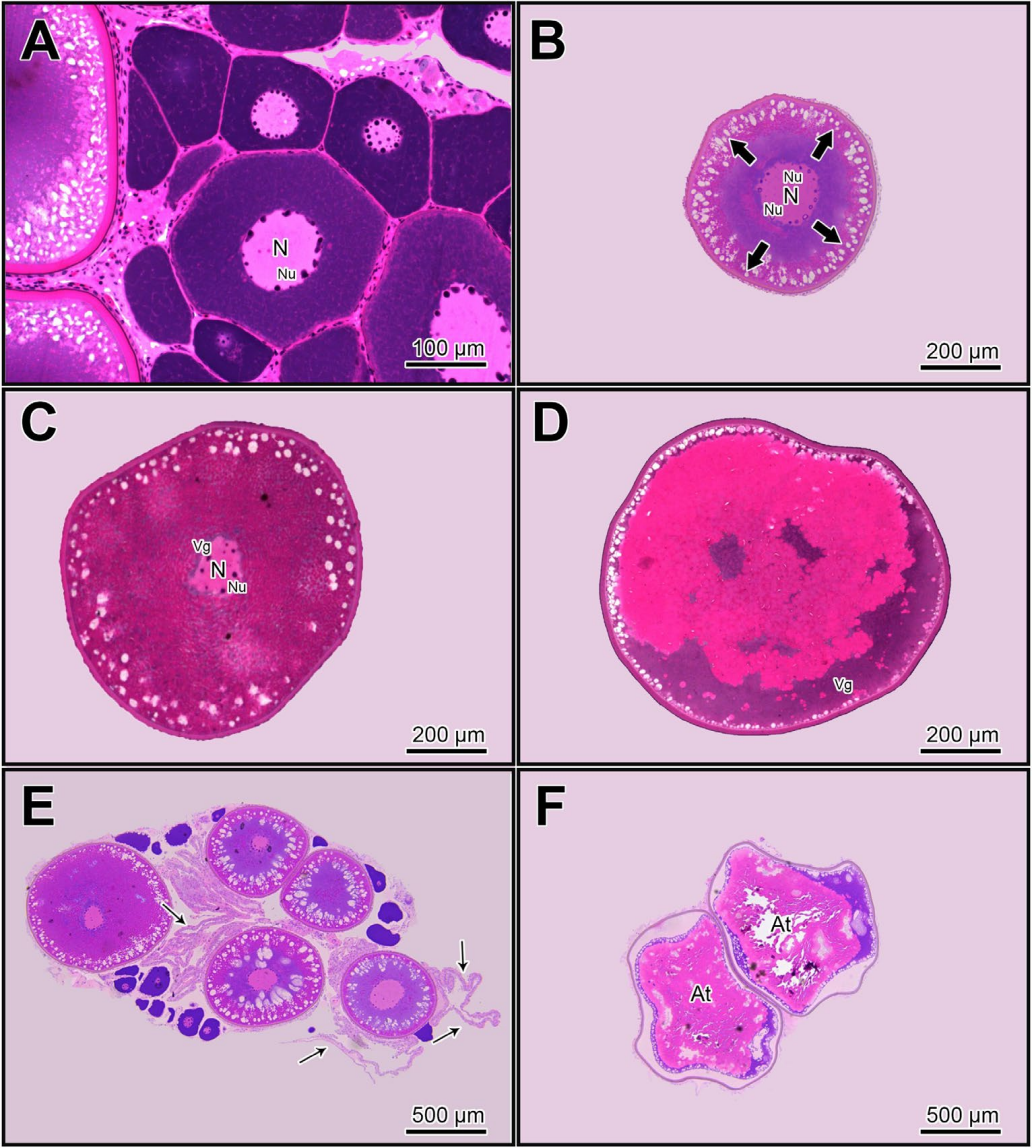
Photomicrographs of histological sections of *Leporinus friderici* oocytes considered for ovary volume density evaluation in this sytudy. (A) Pre-vitelogenic (PV) (270.1 ± 8.8 μm); (B) Cortical alveolus oocytes (CA) (478.4 ± 25.0 µm) with prominent alveolos at cell border (thick arrows); (C) Complete vitelogenic oocytes (CV) (692.5 ± 35.9 µm); (D) Oocytes that remained attached to the ovaries, showing germinal vesicle break down (GVBD oocytes) ruptured at the periphery of the cell (Vg); (784.3 ± 29.4 µm); (E) Post-ovulatory follicles (thin arrows); (F) Atretic oocytes (AT). N: nucleus; Nu: Nucleoli.

The morphological characteristics of oocytes considered for this evaluation are shown in Figure 1.

#### Experiment 1 - CPE

**Figure 2:**
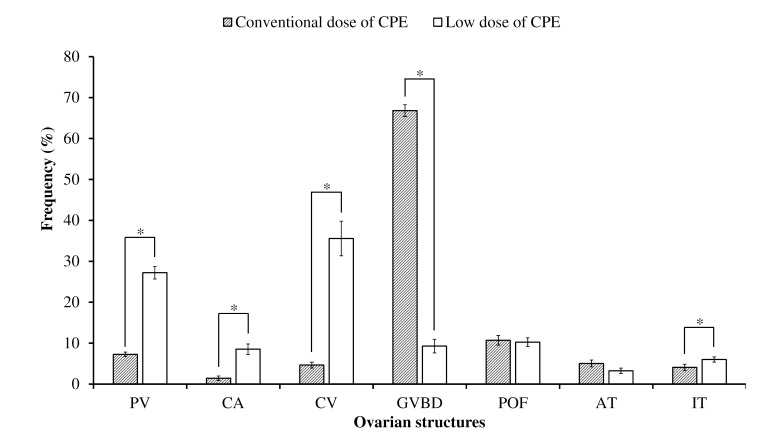
Average percentage values (± SE) of the volume density of different structures within ovaries of *Leporinus friderici* submitted to conventional (0.5 and 5.5 mg/kg) and low (0.5 mg and 1.0 mg/kg) crude carp pituitary extract (CPE) dose, collected just after spawning. Asterisks indicate significant difference between the percentage of the same structure between treatments (p < 0.05). PV: Pre-vitellogenic; CA: Cortical Alveolus; CV: Complete Vitelogenic; GVBD vitellogenic oocyte with germinal vesicle breakdown; POF: post-ovulatory follicle; AT: atretic oocytes; IT: interstitial tissue.

The average values of GVBD oocytes were remarkably higher (7.6 x) for conventional dose (66.8 ± 1.4%) than for lower dose (9.3 ± 1.7%) (p < 0.05). Value of POF were similar between treatments (p < 0.05). The average values of PV (7.3 ± 0.6% and 27.2 ± 1.5%), CA (1.4 ± 0.6 and 8.5 ± 1.3%), CV (4.7 ± 0.7% and 35.6 ± 4.2%) and IT (4.1 ± 0.8% and 6.0 ± 0.6%) were lower for conventional dose than for lower dose (p < 0.05) (Figure 2).

#### Experiment 2 - mGnRHa +MET

**Figure 3:**
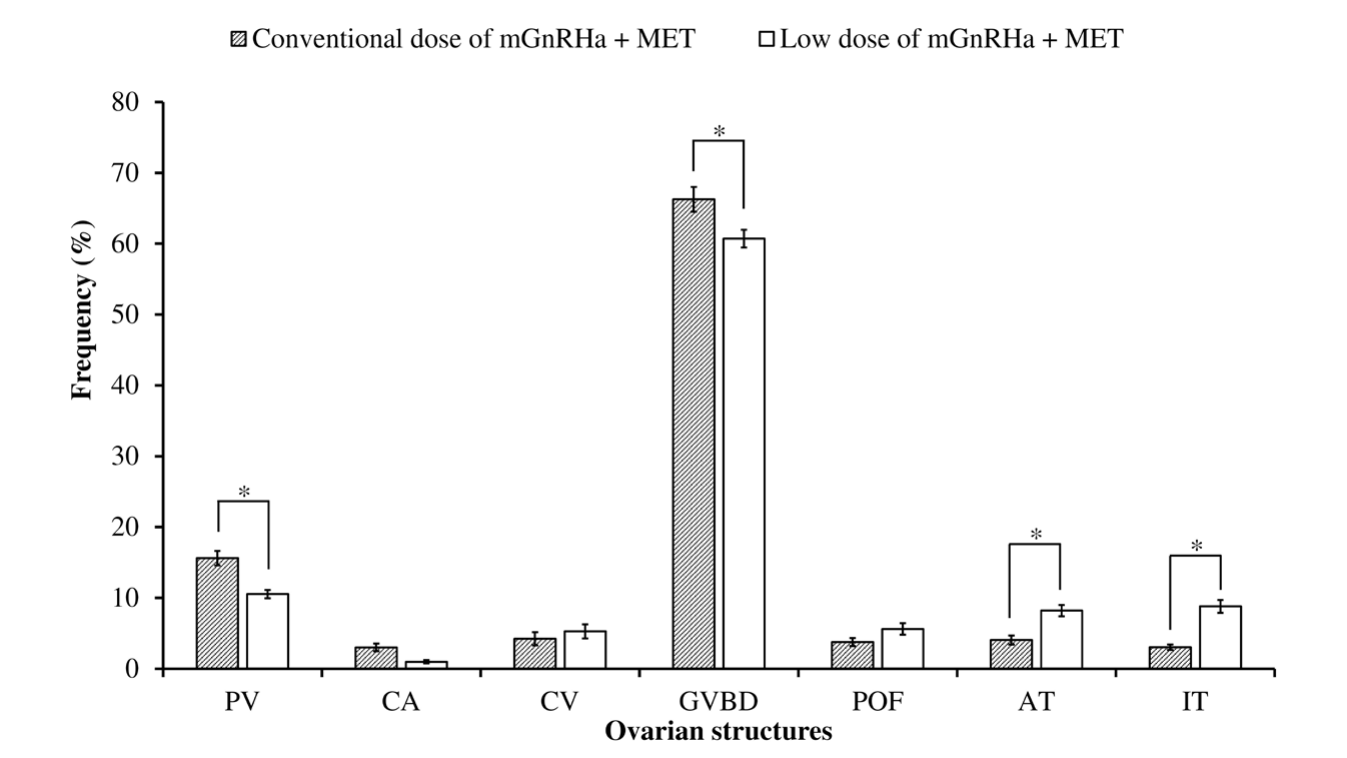
Average percentage values (± SE) of the volume density of different structures within ovaries of *Leporinus friderici* submitted to conventional (40 µg + 20 mg mGnRHa MET/kg (single dose)) and low (4 μg mGnRHa + 2 mg MET (first dose) and 8 µg + 4 mg of mGnRHa + MET (second dose)/kg) dose, collected just after spawning. Asterisks indicate significant difference between the percentage of the same structure between treatments (p < 0.05). PV: Pre-vitellogenic; CA: Cortical Alveolus; CV: Complete Vitelogenic; GVBD vitellogenic oocyte with germinal vesicle breakdown; POF: post-ovulatory follicle; AT: atretic oocytes; IT: interstitial tissue.

The mean values of PV (15.6 ± 1.0% and 10.5 ± 0.6%) and GVBD oocytes (66.3 ± 1.8% and 60.7 ± 1.3%) were significantly higher for conventional dose than for lower dose (p < 0.05). However, mean AT (4.1 ± 0.6% and 8.2 ± 0.8%) and IT (3.0 ± 0.4% and 8.8 ± 0.9%) values were significantly lower for conventional than for lower dose (p < 0.05). The volume densities of POF were similar between treatments (p > 0.05) (Figure 3).

### Gonadal steroids

#### Experiment 1 - CPE

**Figure 4:**
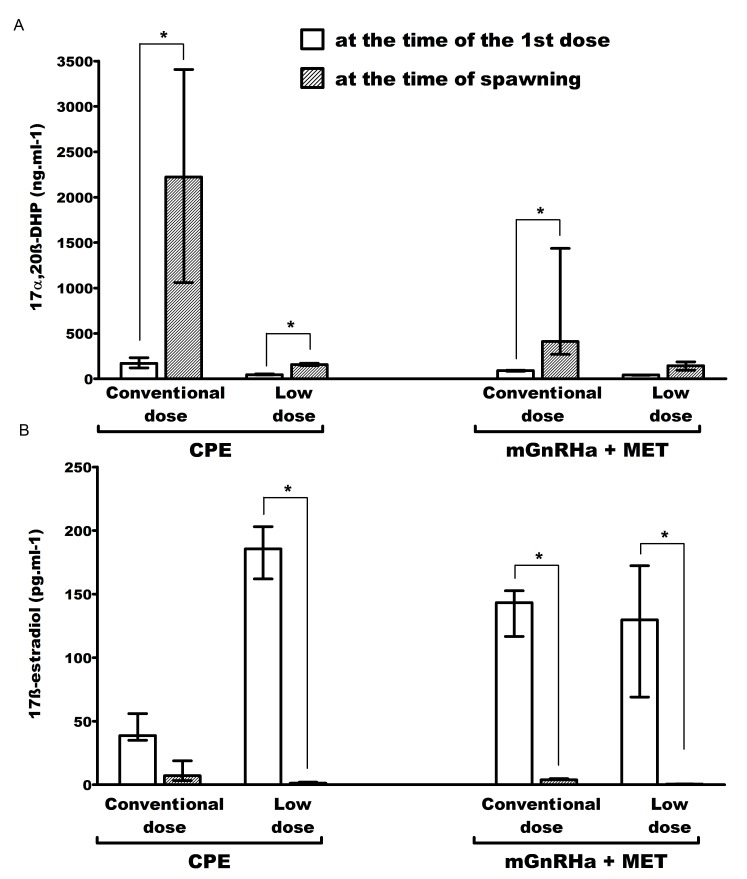
(A) Plasma concentrations of 17α-20β-dihydroxy-4-pregnen-3-one and; (B) 17β-estradiol of *Leporinus friderici* submitted to conventional (0.5 and 5.5 mg/kg) and low (0.5 mg and 1.0 mg/kg) crude carp pituitary extract (CPE) dose and to conventional (40 µg + 20 mg/kg) and low (4 μg + 2 mg and 8 µg + 4 mg/kg) mGnRHa + MET dose. *Significant difference between the same groups in different periods of evaluation.

For the CPE conventional and lower dose, the concentrations of DHP were respectively 13 and 3.5 times increased between the time of first (or single) dose (170.0 ng/mL, 120.7-233.3 and 46.2 ng/mL, 44.2-56.1) and at the time of ovulation (2,224.0 ng/mL, 1060.6-3407.2 and 158.0 ng/mL, 146.9-171.4) (Figure 4).

Regarding E_2_, the values remained stable for conventional dose treatment, but the medians were 143 times lower at the time of first dose (185.6 pg/mL, 162.1-203.1) in comparison to the time of ovulation (1.3 pg/mL, 0.8-2.1) for lower dose treatment (Figure 4).

#### Experiment 2 – mGnRHa + MET

For mGnRHa + MET conventional dose, DHP medians increased 4.6 times from the time of first dose (90.6 ng/mL, 84.2-95.6) in comparison to the time of ovulation (412.4 ng/mL, 270.1-1,436.1). On the other hand, the treatment with lower mGnRHa + MET dose presented stable medians comparing the two analysis times (Figure 4).

Concerning E_2_ concentrations, medians decreased 35.8 times from the time of the first hormonal dose (143.3 pg/mL, 116.7-152.6) in comparison to the time of ovulation (3.9 pg/mL, 3.9-4.9) for the conventional dose treatment. In the lower dose treatment, the median values of E_2_ were 272 times reduced (from 129.8 pg/mL, 69.1-172.3 to 0.5 pg/mL, 0.4-0.7) (Figure 4).

## Discussion

In this study we observed that CPE and mGnRHa + MET treatments provoked ovulation in *L. friderici*, but the lower CPE dose treatment was the unique that provided viable embryos. This pattern of response is unusual mainly because conventional CPE dose (0.5 mg and 5.5 mg/kg, with 12h interval, with some variations) has provided ovulation and viable embryos in many fish species evaluated worldwide (Mylonas et al., 2010) and is the mainly hormonal therapy used for inducing tropical migratory fish ovulation (Caneppele et al., 2015; Souza et al., 2015; Ittzés et al., 2015; Viveiros et al., 2015; Sanches et al., 2016; Pereira et al., 2017; García et al., 2017).

However, the consistency in the results obtained using CPE and mainly mGnRHa in South American migratory fish is very far from being a reality. Although recurrent in fish farms, unfortunately the failures that occur with the use of CPE and GnRH protocols for induced breeding in this species are rarely reported and specific scientific studies are scarce (Ramos et al., 1997; Acuña and Rangel, 2009; Paulino et al., 2011; Criscuolo-Urbinati et al., 2012; Hainfellner et al., 2012a; Pereira et al., 2017). The heterogeneous results concerning fertility rates and the considerable number of oocytes that are retained in the post-spawning ovaries using CPE (0.9 and 5.9 CPE/kg, 14 h interval) are highlighted by Sato et al. (2000) in a congener species, *Leporinus elongatus*. A high proportion of oocytes retained in the ovaries after stripping (week ovulation) also seems to be a constant in treatments using conventional CPE protocols in other South American rheophilic species (Hainfellner et al., 2012a; Sato et al., 2000; Criscuolo-Urbinati et al., 2012; Pereira et al., 2017). In the present study, even in the treatment with lower CPE dose that presented the best results, 42% of post spawned ovaries volume was made up by mature vitellogenic oocytes (33%) and GVBD oocytes (9%), corroborating that also for *L. friderici* the CPE treatments can provoke ovulation but a large number of oocytes may remain unovulated in the ovaries.

The foundation for the use of fractioned CPE dose was demonstrated in carps decades ago, in which GVBD and ovulation only occurs in fractioned carp pituitary extract (0.07 mg and 0.35 mg carp gonadotrophin/kg body weight, 11 h interval) treated- females. On the other hand, in ovaries of females treated with only the resolving dose (0.35 mg carp gonadotrophin/kg body weight), GVBD and ovulation are not observed (Levavi-Zermonsky and Yaron, 1986). From that point, when an association between successful ovulation and the fractionation of the CPE protocol was established, no further studies focused on the resolving dose concentration able to provide the best ovulation rate together with viable embryos, for any tropical migratory rheophilic fish species. In that regard, considering that in the present study the priming CPE dose was similar for both treatments, if in one hand the resolving dose of both treatments were associated with similar POF volume density and similar fecundity rates, on the other hand, the resolving dose of the conventional treatment was associated with 100% death of embryos. This observation alone, already shows that the conventional CPE protocol is not suitable for this species. Our results indicated that future studies involving a higher number of doses considering ovulation rates and survival of embryos are necessary to develop a more efficient protocol.

The main evidence for this conclusion comes from the fact that in the lower-dose CPE treatment, the percentage of CV oocytes, in post-spawning ovaries, was lower than that of the conventional CPE treatment, indicating that the former may have provoked a less intense GVBD. On the other hand, conventional CPE treatment was associated to a markedly higher proportion of GVBD oocytes indicating that this treatment may have been less effective in inducing ovulation. Taken together this observation points out to the possibility of determining a more efficient protocol where the best GVBD and ovulation rates can occur together with the best embryo surveillance rates.

Regarding the results obtained with the use of mGnRHa + MET, both failed to provide viable embryos, and in addition, both presented spawning and relative fecundity rates much lower than the CPE treatments. Therefore, the mGnRHa + MET treatments applied here cannot be considered suitable both for inducing ovulation and for providing viable embryos, even when applied in lower concentration. In this context, some previous studies have reported that the use of synthetic products causes ovulation in native migratory fish but fails to provide viable embryos, probably due to treatment toxicity (Pereira et al., 2017). The potential use of GnRH in tropical migratory species was initially showed by Carolsfeld et al. (1988) showing that LHRH ethylamide (100 µg/kg in single dose) can provoke final maturation and ovulation in *Piaractus mesopotamicus*, another tropical reophilic fish. However, the use of GnRH or other synthetic products was not continued due to inconsistent results obtained in later studies with the same species (Paulino et al., 2011), as well as for other South American reophilic species (Ramos et al., 1997; Carneiro and Mikos, 2008; Acuña and Rangel, 2009; Paulino et al., 2011; Pereira et al., 2017).

Concerning gonadal steroids, except for the lower mGnRHa + MET dose, we observed that the concentrations of DHP, the main substance inducing the final maturation and ovulation in fish (Klangnurak and Tokumoto, 2017; Honji and Moreira, 2017), increased ​​between the time of first hormonal dose and the moment of the ovulation in all other treatments. This aspect was apparently associated with the fact that in the lower mGnRHa + MET only 50% of the females effectively spawned. Conventional CPE treatment, despite having a prominent elevation in DHP values at the time of ovulation ​​ (13x higher than the values ​​of the first dose), presented spawning rates similar to the low CPE dose (where this increase was only 3.47x). These findings indicate that there is a hypothetically adequate concentration of DHP to be established in future approaches using CPE that promote successful ovulation together with viable embryos, specially concerning wild broodstocks.

Still concerning the experiment with CPE, we observed a reduction of E_2_ concentrations between the time of first hormonal dose and the moment of ovulation in all treatments, except for the conventional CPE treatment. The values were relatively low in all treatments at the time of ovulation, with medians varying from 0.5 to 7.2 pg/mL, when compared to the values observed at the time of the first dose (with medians ranging from 38.8 to 185.6 pg/mL). These observations pointed out that ovulation was associated with reductions of E_2_ in almost all treatments characterizing a phenomenon known as steroidogenic shift known to be associated to successful ovulation with a concomitant and respectively increase and decrease of DHP and E_2_ concentrations during induced spawning (Levavi-Zermonsky and Yaron, 1986; Nagahama and Yamashita, 2008; Podhorec et al., 2016; Klangnurak and Tokumoto, 2017; Honji and Moreira, 2017).

Taking together, for the CPE experiment, the apparently high levels of DHP (2,224.0 ng/mL of conventional vs. 158.0 ng/mL of lower CPE dose) and E_2_ (7.2 pg/mL of conventional against 1.3 pg/mL of the lower CPE dose) of conventional dose treatment at ovulation might be associated to the mortality of the embryos. The data obtained here indicate that *in vitro* studies exposing eggs and oocytes of these species to different concentrations of gonadal steroids may indicate a clearer toxicity scenario.

Concerning the steroid levels of the mGnRHa + MET experiment, since no viable embryos were found in any of the doses applied, no conclusions can be made on the relationship between the concentrations of gonadal steroids and the embryonic mortality. However, apparently some substance or derivative present in the treatment itself (other than gonadal steroids measured) may have caused toxicity to the embryos, similarly to recent results described for a congener, *L. macrocephalus* (Pereira et al., 2017). On the other hand, in one of the species studied, it has been shown that very low concentrations of GnRH (1 µg of mGnRHa/kg) may be effective in inducing ovulation in some species (Podhorec et al., 2011). Thus, it is possible that the use of even lower dose of GnRH than the one we used here, or other combinations (such as with other dopamine inhibitors) at relatively low concentrations, still need to be tested in this species for a more conclusive idea in this concern.

In recent years, several studies have been developed to support the replacement of CPE by synthetic products for induced reproduction of tropical migratory fish. However, the inconsistency and non-reproducibility of the results have made the use of these products a distant reality. Induced ovulation with Ovaprim^®^ (single dose: 10 µg Salmon Gonadotropin Releasing hormone analog (sGnRHa)/kg + 5 mg domperidone/kg) was well succeeded in *Colossoma macropomum*, but provided lower quality embryos when compared to CPE conventional dose (Acuña and Rangel, 2009). In a congener species, *L. macrocephalus*, lower dose of mGnRHa (7 μg mGnRH + 10 mg MET/kg) provoked ovulation, but not viable embryos (Pereira et al., 2017). The induced ovulation failed in *Rhamdia quelen* (two doses: 2 µg mGnRHa + 1 mg MET/kg and 20 µg mGnRHa + 10 mg MET/kg) (Carneiro and Mikos, 2008). When applied to *Piaractus mesopotamicus*, *Brycon orbygnianus* and *Prochilodus lineatus,* busserelin acetate caused ovulation in all three species, but no viable embryos were obtained after fertilization (Paulino et al., 2011). Taken together, these findings indicate that *L. friderici*, as well as other South American migratory species, has probably a relatively high sensibility (due to completely unknown reasons) to hormonal induction, especially for GnRH, which provides inconstant and very heterogeneous results of difficult reproducibility. Mainly considering that lower dose applied here (4 μg mGnRHa + 2 mg MET/kg and 8 µg mGnRHa + 4 mg MET/kg) were much lower than GnRHa dose reported as causing overstimulation for other fish species (50 μg/kg) (Mañanos et al., 2002; Rosenfeld et al., 2012).

In summary, lower CPE dose is promising for obtaining ovulation and viable *L. friderici* embryos, however, conventional and lower mGnRHa + MET dose applied here produced ovulation at lower intensities but failed to provide viable embryos. The evaluation of the kinetics of the meiotic process observed for both CPE treatments indicates that the lower dose protocol, which presented better results, can be further optimized since, in spite of inadequate, the conventional dose protocol presented a higher potential to induce GVBD then the lower dose treatment. We thus corroborate the need for specific studies on the establishment of effective ovulation induction protocols for this and other species, by means of specific studies, evaluating the association between concentration, number of doses (as well as the interval between them), the evolution of the meiotic process, as well as the possible cytotoxic effects of the hormones and by-products generated on the oocytes and eggs.

## Conclusion

Among the treatments applied, the only one that generated viable *Leporinus friderici* embryos was the protocol with reduced doses of CPE (0.5 and 1.0 mg/kg). All treatments provoked final maturation and ovulation. Comparing to reduced doses of CPE protocol, the conventional CPE protocol (0.5 and 5.5 mg/kg) was more effective in promoting final maturation (GVBD), however most of these oocytes remained retained in the ovaries, showing an imbalance between final maturation and ovulation. Except for the protocol with low dose of mGNRHa, we observed an increase in DHP between the beginning of hormonal treatment and ovulation in all treatments. The most intense increase in DHP was observed in the treatment with a conventional dose of CPE, but it was neither associated with a better rate of POF nor viable embryos. Our data showed that wild breeders of this species respond better to treatments with reduced doses of CPE.

## List of abbreviations

AT: atretic oocyte
ATU: accumulated thermal units
CA: cortical alveoli oocyte
CPE: carp pituitary extract
CV: complete vitellogenic oocyte
DHP: 17α,20β-dihydroxy-4-pregnen-3-one
E2: 17β estradiol
FS: fertilization success
Fsh: Follicle Stimulating Hormone
GnRHa: Gonadotrophin-releasing hormones analogs
GVBD: germinal vesicle break down
GVBD oocytes: mature vitellogenic oocytes with cytoplasm filled entirely by yolk and showing germinal vesicle break down
hpf: hours post-fertilization
HS: hatching success
IBGE: Instituto Brasileiro de Geografia e Estatística
IT: Interstitial tissue
LH: Luteinizing Hormone
LHRH: luteinizing hormone-releasing hormone
MET: metoclopramide
mGnRHa: mammalian GnRH analogue
POF: post-ovulatory follicles
PV: previtellogenic oocyte
RF: relative fecundity
sGnRHa: Salmon Gonadotropin Releasing hormone analogue
SR: spawning rate

## References

[B001] Acuña JJA, Rangel JLH (2009). Effects of hypophyseal extract of common carp and the analogue of the GnRH on the final maturation oocyte and the spawning of cachama negra (*Colossoma macropomum*). Rev Cient.

[B002] Andrade DR, Souza GAP, Vidal MV (2005). Morfometria como instrumento da estimação da fecundidade de fêmeas de piau-vermelho (*Leporinus copelandii*), na bacia do Baixo Rio Paraíba do Sul. Rev Ceres.

[B003] Bobe J, Labbé C (2010). Egg and sperm quality in fish. Gen Comp Endocrinol.

[B004] Borella MI, Chehade C, Costa FG, Batlouni SR, Baldisserotto B, Cyrino JEP, Urbinati EC (2014). Mecanismo de desenvolvimento ovocitário em peixes de água doce utilizados em aquicultura. Biologia e fisiologia de peixes neotropicais de água doce.

[B005] Borella MI, Gomes CC, Costa FG, Jesus LWO, Cassel M, Batlouni SR, Baldisserotto B, Cyrino JEP, Urbinati EC (2019). The brain-pituitary-gonad axis and the gametogenesis. Biology and physiology of freshwater neotropical fish.

[B006] Brasil (2014). 1º Anuário Brasileiro da Pesca e Aquicultura.

[B007] Brown M, Robinson C, Davies IM, Moffat CF, Redshaw J, Craft JA (2004). Temporal changes in gene expression in the liver of male plaice (*Pleuronectes platessa*) in response to exposure to ethynyl oestradiol analysed by macroarray and Real-Time PCR. Mutat Res Genet Toxicol Environ Mutagen.

[B008] Caneppele D, Sanches EA, Romagosa E (2015). Sperm production of *Steidachneridion parahybae* (Steindachner 1877) and the effect of hormonal induction throughout one reproductive cycle. J Appl Ichthyology.

[B009] Carneiro PCF, Mikos JD (2008). Gonadotrofina coriônica humana e hormônio liberador de gonadotrofina como indutores da reprodução do jundiá. Acta Sci Anim Sci.

[B010] Carolsfeld J, Ramos SM, Ormanezi R, Gomes JH, Barbosa JM, Harvey B (1988). Analysis of protocols for application of an LHRH analog for induced final maturation and ovulation of female pacu (*Piaractus mesopotamicus*). Aquaculture.

[B011] Cejko BI, Targońska K, Kowalski RK, Żarski D, Sarosiek B, Kucharczyk D, Glogowski J (2012). The effectiveness of hormonal preparations (Ovopel, Ovaprim, LHRHa, hCG and CPE) in stimulating spermiation in dace *Leuciscus leuciscus* (L.). J Appl Ichthyology.

[B012] Criscuolo-Urbinati E, Kuradomi RY, Urbinati CE, Batlouni SR (2012). The administration of exogenous prostaglandin may improve ovulation in pacu (*Piaractus mesopotamicus*). Theriogenology.

[B013] García S, Yasui GS, Bernardes-Júnior JJ, Silva BC, Amaral-Júnior H, Zaniboni-Filho E (2017). Induction of triploidy in *Rhamdia quelen* (Siluriformes, Heptapteridae) by double-temperature shock. Lat Am J Aquat Res.

[B014] Hainfellner P, Kuradomi RY, de Souza TG, Sato RT, Figueiredo-Ariki DG, de Freitas GA, Queiroz L, Valenti WC, Valenti PM, Ge W, Batlouni SR (2019). Reproductive cycle of the Amazonian planktivorous catfish *Hypophthalmus marginatus* (Siluriformes, Pimelodidae). Aquacult Res.

[B015] Hainfellner P, Souza TG, Munõz ME, Freitas GA, Batlouni SR (2012). Spawning failure in *Brycon amazonicus* may be associated with ovulation and not with final oocyte maturation. Arq Bras Med Vet Zootec.

[B016] Hainfellner P, Souza TG, Moreira RG, Nakaghi LSO, Batlouni SR (2012). Low estradiol levels, delayed vitellogenesis and reduced amounts of yolk are dysfunctions associated with the formation of low quality oocytes in *Prochilodus lineatus* (Teleostei: characiformes). Neotrop Ichthyol.

[B017] Honji RM, Moreira RG (2017). Controle neuroendócrino da ovogênese em peixes teleósteos. Rev Bras Reprod Anim.

[B018] IBGE (2018). Produção da pecuária municipal.

[B019] Ittzés IT, Szabó EK, Kronbauer EC, Urbányi B (2015). Ovulation induction in jundia (*Rhamdia quelen*, Heptapteridae) using carp pituitary extract or salmon GnRH analogue combined with dopamine receptor antagonists. Aquacult Res.

[B020] Klangnurak W, Tokumoto T (2017). Fine selection of up-regulated genes during ovulation by in vivo induction of oocyte maturation and ovulation in zebrafish. Zoological Lett.

[B021] Kuradomi RY, Batlouni SR (2018). PGF2α and gonadal steroid plasma levels of successful and unsuccessful spawning *Piaractus mesopotamicus* (Teleostei, Characiformes) females. Aquacult Int.

[B022] Levavi-Zermonsky B, Yaron Z (1986). Changes in gonadotropin and ovarian steroids associated with oocytes maturation during spawning induction in the carp. Gen Comp Endocrinol.

[B023] Lopes JP, Leal ALG (2010). Desova induzida em Piau-de-vara *Schizodon fasciatus* spix & agassiz, 1829 para propagação artificial. Revista Brasileira de Engenharia de Pesca.

[B024] Mañanos E, Carrillo M, Sorbera LS, Mylonas CC, Asturiano JF, Bayarri MJ, Zohar Y, Zanuy S (2002). Luteinizing hormone and sexual steroid plasma levels after treatment of European sea bass with sustained-release delivery systems for gonadotropin-releasing hormone analogue. J Fish Biol.

[B025] Mylonas CC, Fostier A, Zanuy S (2010). Broodstock management and hormonal manipulations of fish reproduction. Gen Comp Endocrinol.

[B026] Nagahama Y, Yamashita M (2008). Regulation of oocyte maturation in fish. Dev Growth Differ.

[B027] Paulino MS, Milliorini AB, Murgas LDS, Lima FSM, Felizardo VO (2011). Desempenho reprodutivo do pacu, piracanjuba e curimba induzidos com extrato de buserelina. Bol Inst Pesca.

[B028] Pereira TSB, Boscolo CNP, Moreira RG, Batlouni SR (2017). The use of mGnRHa provokes ovulation but not viable embryos in *Leporinus macrocephalus.*. Aquacult Int.

[B029] Podhorec P, Socha M, Ammar IB, Sokolowska-Mikolajczyk M, Brzuska D, Milla S, Gosiewski G, Stejskal V, Simko M, Kouril J (2016). The effects of GnRHa with and without dopamine antagonist on reproductive hormone levels and ovum viability in tench *Tinca tinca.*. Aquaculture.

[B030] Podhorec P, Socha M, Sokolowska-Mikolajczyk MB, Drozd T, Policar T, Stejskal V, Kouril J (2011). Effective dose of mGnRHa for induction of ovulation in tench (*Tinca tinca* L.). Aquaculture.

[B031] Ramos RO, Ramos SM, Mendonça JOJ (1997). Utilização de análogos do LHRH na indução à ovulação do Matrinchã, *Brycon cephalus. (GÜNTHER, 1869).*. Bol Tec CEPTA.

[B032] Rosenfeld H, Mylonas CC, Bridges CR, Heinisch G, Corriero A, Vassallo-Aguis R, Medina A, Belmonte A, Garcia A, De la Gándara F, Fauvel C, De Metrio G, Meiri-Ashkenazi I, Gordin H, Zohar Y (2012). GnRHa-mediated stimulation of the reproductive endocrine axis in captive Atlantic bluefin tuna, *Thunnus thynnus.*. Gen Comp Endocrinol.

[B033] Sanches EA, Caneppele D, Okawara RY, Damasceno DZ, Bombardelli RA, Romagosa E (2016). Inseminating dose and water volume applied to the artificial fertilization of *Steindachneridion parahybae* (Steindachner, 1877) (Siluriformes: Pimelodidae): Brazilian endangered fish. Neotrop Ichthyol.

[B034] Sato Y, Fenerich-Verani N, Verani JR, Vieira LJS, Godinho HP (2000). Induced reproductive responses of the neotropical anostomid fish *Leporinus elongatus* under captive breeding. Aquacult Res.

[B035] Schorer M, Moreira RG, Batlouni SR (2016). Selection of pacu females to hormonal induction: effect of age and evaluation methods. Bol Inst Pesca.

[B036] Souza FN, Fatima FME, Corrêa RAC, Abreu JS, Pires LB, Streit DP, Lopera-Barrero NM, Povh JA (2018). Ovopel^®^ and carp pituitary extract for induction of reproduction in *Colossoma macropomum* females. Anim Reprod Sci.

[B037] Souza TG, Hainfellner P, Kuradomi RY, Muñoz ME, Honji RM, Moreira RG, Batlouni SR (2015). Inappropriate management conditions, especially for the regressed class, are related to sperm quality in *Prochilodus lineatus.*. Theriogenology.

[B038] Vaz MM, Torquato VC, Barbosa NDC (2000). Guia ilustrado de peixes da bacia do Rio Grande.

[B039] Viveiros ATM, Gonçalves ACS, Di Chiacchio IM, Nascimento AF, Romagosa E, Leal MC (2015). Gamete quality of streaked prochilod *Prochilodus lineatus* (Characiformes) after GnRHa and dopamine antagonist treatment. Zygote.

[B040] Von Ihering R, Azevedo P (1936). A desova e a hipofisação dos peixes: evolução de dois Nematognathas. Arq Inst Biol.

